# Inhaled Steroids Modulate Extracellular Matrix Composition in Bronchial Biopsies of COPD Patients: A Randomized, Controlled Trial

**DOI:** 10.1371/journal.pone.0063430

**Published:** 2013-05-07

**Authors:** Lisette I. Z. Kunz, Jolanda Strebus, Simona E. Budulac, Therese S. Lapperre, Peter J. Sterk, Dirkje S. Postma, Thais Mauad, Wim Timens, Pieter S. Hiemstra, the GLUCOLD (Groningen Leiden Universities Corticosteroids in Obstructive Lung Disease) Study Group

**Affiliations:** 1 Department of Pulmonology, Leiden University Medical Center, Leiden, The Netherlands; 2 Department of Epidemiology, University of Groningen, University Medical Center Groningen, Groningen, The Netherlands; 3 Department of Respiratory Medicine, Academic Medical Center, Amsterdam, The Netherlands; 4 Department of Pulmonology, University of Groningen, University Medical Center Groningen, Groningen, The Netherlands; 5 Department of Pathology, São Paulo University Medical School, São Paulo, Brazil; 6 Department of Pathology, University of Groningen, University Medical Center Groningen, Groningen, The Netherlands; Leiden University Medical Center, The Netherlands

## Abstract

**Rationale:**

Smoking and inflammation contribute to the pathogenesis of chronic obstructive pulmonary disease (COPD), which involves changes in extracellular matrix. This is thought to contribute to airway remodeling and airflow obstruction. We have previously observed that long-term treatment with inhaled corticosteroids can not only reduce bronchial inflammation, but can also attenuate lung function decline in moderate-severe COPD. We hypothesized that inhaled corticosteroids and current smoking modulate bronchial extracellular matrix components in COPD.

**Objective:**

To compare major extracellular matrix components (elastic fibers; proteoglycans [versican, decorin]; collagens type I and III) in bronchial biopsies 1) after 30-months inhaled steroids treatment or placebo; and 2) between current and ex-smokers with COPD.

**Methods:**

We included 64 moderate-severe, steroid-naive COPD patients (24/40 (ex)-smokers, 62±7 years, 46 (31–54) packyears, post-bronchodilator forced expiratory volume in one second (FEV1) 62±9% predicted) at baseline in this randomized, controlled trial. 19 and 13 patients received 30-months treatment with fluticasone or placebo, respectively. Bronchial biopsies collected at baseline and after 30 months were studied using (immuno)histochemistry to evaluate extracellular matrix content. Percentage and density of stained area were calculated by digital image analysis.

**Results:**

30-Months inhaled steroids increased the percentage stained area of versican (9.6% [CI 0.9 to 18.3%]; p = 0.03) and collagen III (20.6% [CI 3.8 to 37.4%]; p = 0.02) compared to placebo. Increased collagen I staining density correlated with increased post-bronchodilator FEV_1_ after inhaled steroids treatment (Rs = 0.45, p = 0.04). There were no differences between smokers and ex-smokers with COPD in percentages and densities for all extracellular matrix proteins.

**Conclusions:**

These data show that long-term inhaled corticosteroids treatment partially changes the composition of extracellular matrix in moderate-severe COPD. This is associated with increased lung function, suggesting that long-term inhaled steroids modulate airway remodeling thereby potentially preventing airway collapse in COPD. Smoking status is not associated with bronchial extracellular matrix proteins.

**Trial Registration:**

ClinicalTrials.gov NCT00158847

## Introduction

Chronic Obstructive Pulmonary Disease (COPD) is characterized by an abnormal inflammatory response and structural alterations of the bronchial wall and parenchyma [1]. This pulmonary remodeling has been linked to airflow limitation in COPD [2], [3]. Changes in the extracellular matrix (ECM), produced by (myo)fibroblasts, epithelial cells and airway smooth muscle cells, contribute to this remodeling process and alter airway mechanics and dynamics [4], [5]. The ECM consists of three major components: elastic fibers, proteoglycans and collagens, which are involved in cell migration, proliferation, adhesion, water balance and regulation of inflammatory mediators [4].

The composition of the pulmonary ECM is different in subjects with and without COPD. Fewer elastic fibers are found in small airways and alveoli of COPD patients than in healthy controls [6], [7]. Furthermore, versican, a large proteoglycan is more abundant, while the small proteoglycan decorin is reduced in small airways in COPD compared to healthy subjects [8]–[10]. Collagens are the main component of the ECM, and collagen composition differs between COPD patients and healthy controls as shown by the observation that collagen type I is lower in the large and small airways [11] and collagen type III expression is lower in the small airways of COPD patients than in healthy controls [3].

Since smoking is a risk factor for COPD, this may also influence ECM composition. Indeed, cigarette smoke has been shown to induce secretion of several profibrotic growth factors, including transforming growth factor-beta (TGF-β), both in human lung fibroblasts and in lung tissue of COPD patients [12], [13]. Rodent models exposed to cigarette smoke had less lung elastic fibers, but more collagens than sham-smoked animals [14]. Others even reported an increased elastic fibers gene expression in lung tissue of severe COPD patients [15]. Smoke exposure decreased proteoglycan expression as demonstrated by a study with pulmonary fibroblasts from moderate and very severe COPD patients [16].

Although generally (neutrophil dominated) inflammation in COPD is considered to be resistant to steroids treatment, we recently observed that long-term inhaled corticosteroids (ICS) treatment partially decreased bronchial inflammation (CD3^+^, CD4^+^, CD8^+^ and mast cells) -without effects on neutrophils- and attenuated lung function decline in moderate-severe COPD patients participating in the GLUCOLD (Groningen Leiden Universities Corticosteroids in Obstructive Lung Disease) study [17]. ICS may affect ECM through various mechanisms, including modulation of inflammation by profibrotic mediators and targeting ECM genes directly. This may explain differences in the effects of steroids that are observed in *in vivo* and *in vitro* studies. Whereas steroid treatment of asthmatics did not change elastic fibers and collagens in bronchial biopsies [18], steroids did inhibit serum-induced proteoglycan production in fetal lung fibroblasts [19]. In contrast to asthma, to the authors’ knowledge, effects of ICS on ECM composition in COPD patients have not been described.

We hypothesized that inhaled steroids treatment modulates bronchial ECM components in COPD. In addition, we hypothesized that current smoking affects bronchial ECM.

## Materials and Methods

### Subjects and Study Design

The current study is a substudy of the GLUCOLD (Groningen Leiden Universities Corticosteroids in Obstructive Lung Disease) study, a double-blind, placebo-controlled randomized trial in which 114 moderate-severe COPD steroid-naive patients were included [17]. The protocol for this trial and supporting CONSORT checklist are available as supporting information; see Checklist S1 and Protocol S1. Clinically stable subjects participating in the GLUCOLD study were aged 45–75 years, smoked ≥10 packyears, were current or ex-smokers with ≥one month of smoking cessation and were allowed to use short-acting bronchodilators. Exclusion criteria were asthma and ICS use in the previous 6 months. Patients were randomly assigned to receive one of four treatments for 30 months: 1) fluticasone propionate 500 µg bid; 2) fluticasone/salmeterol 500/50 µg bid; 3) fluticasone 500 µg bid (6 months) and followed by placebo (24 months); or 4) placebo bid. Diskus dry-powder inhalers (GlaxoSmithKline, Zeist, The Netherlands), were used for inhalation of the study medication and placebo, and both had equal appearance. For the current study we used tissue and data of group 1 and 4. Spirometry, reversibility to salbutamol and airway hyperresonsiveness (PC_20_) were determined according to international guidelines [20], [21]. Approval of the medical ethics committees of both centers was obtained: all subjects provided written informed consent [17].

### Bronchoscopy and Bronchial Biopsies

A fiberoptic bronchoscopy was performed at baseline and after 30 months according to standardized protocols [22]. Six bronchial biopsies per patient per visit were collected at the 3^rd^–5^th^ bronchial level, one with the best morphology being used. Tissue of 64 out of 114 patients was available due to use in previous studies [17], [22], [23].

### (Immuno)Histochemical Stainings

**Figure 1:**
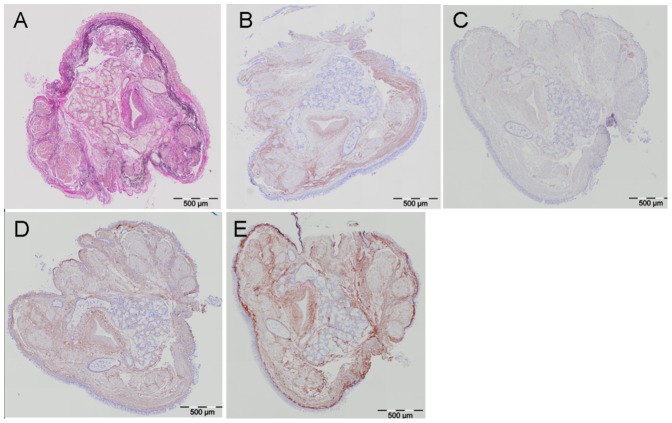
Examples of (immuno)histochemical stainings. The same bronchial biopsy section is shown for the histochemical staining for elastic fibers (A) and the immunohistochemical stainings for versican (B), decorin (C), collagen type I (D) and collagen type III (E). Original magnification 200×.

Processing and analysis of bronchial biopsies was performed in line with the recommendations of the ATS/ERS task force [24] by using an internal reference parameter in the analysis. We did not take specific precautions to orientate the samples during processing to assure that the orientation of the biopsies is randomized [24]. However, since biopsies tend to curl after sampling, a random orientation of the tissue structures is favored during embedding [25]. Sections of 4 µm thickness of paraffin-embedded bronchial biopsies were used for histochemistry (elastic fibers) and immunohistochemistry for proteoglycans and collagens. Elastic fibers were stained according to Weigert’s protocol [26]. Versican, decorin, collagen I and III antibodies were used after appropriate antigen retrieval, followed by horseradish peroxidase-conjugated anti-mouse or anti-rabbit EnVision system (DAKO, Glostrup, Denmark) and the chromogen NovaRed (Vector, Burlingame, CA). Images of stained biopsies are presented in figure 1, and additional information on the stainings is provided in table S1 in file S1.

### Digital Image Analysis

Tissue samples were analyzed in a blinded manner by independent observers, unaware of the subjects’ clinical data (LK, JS). Total biopsy images were acquired using a color camera (200× magnification) and analyzed with image analysis software (CellD, Olympus, Zoeterwoude, The Netherlands). The lamina propria was selected per biopsy (minimum area 0.09 mm^2^). The percentage stained area for a specific ECM component was calculated dividing the stained area by the total selected area (volume fraction; used as an internal reference parameter; [24]). Staining intensity was further analyzed by densitometry (weighted mean per biopsy) and presented as gray value (black: gray value = 0; white: gray value = 255). Only immunohistochemical stainings can represent density, therefore density was not calculated for elastic fibers. Additional information on digital image analysis is provided in file S1.

### Statistical Analysis

Only biopsies from compliant subjects using ≥70% of the prescribed dose were analyzed (per-protocol analysis). Means with standard deviations (SD) and 95% confidence intervals (CI) or medians with interquartile range (IQR) are presented. Differences between smokers and ex-smokers were explored using Mann-Whitney tests. Paired and independent t-tests were used for evaluating the effect of ICS on ECM proteins within and between treatments, respectively. Correlations were analyzed using Spearman correlation coefficient (Rs). Statistical analysis was performed with SPSS 17.0 software (SPSS Inc., Chicago, IL). Significance was inferred at P≤0.05.

## Results

### Patient Characteristics

At baseline, bronchial biopsies of 64 of 114 unselected moderate-severe COPD patients [24/40 (ex-)smokers] were included. A flow diagram of our study is presented in the figure S1. Patient characteristics of the whole group have previously been published [17], [22], [27]. 33 Patients were treated with either fluticasone or placebo for 30 months (19/19 and 13/14 adherent in fluticasone and placebo group, respectively). Mean post-bronchodilator FEV_1_ was 62% predicted (SD 9.9%). Ex-smokers were older at baseline compared to current smokers, as is shown in table 1. Baseline characteristics of the entire group, groups with available and unavailable bronchial biopsies, and the number of available biopsies were not significantly different between both treatment arms. During the study, six patients changed their smoking habits (balanced among groups).

**Table 1:** Patient characteristics at baseline.

	Smokers(n = 40)	Ex-smokers (n = 24)	Placebo(n = 13)	Fluticasone (n = 19)
Males [n (%)]	37 (92.5)	23 (95.8)	12 (92.3)	17 (89.5)
Age (years)	60.9 (7.2)	65.1 (6.6)*	62.5 (7.9)	62.0 (7.4)
Current/ex-smoker (n)			9/4	11/8
Packyears	46.8 (30.9–55.0)	37.5 (32.1–52.5)	42.0 (28.4–58.0)	44.9 (31.2–51.0)
Smoking cessation (years)		5.5 (1.3–10.0)	0.0 (0.0–1.5)	0.0 (0.0–5.0)
FEV_1_ post-bronchodilator (l)	2.05 (0.44)	1.94 (0.46)	1.95 (0.61)	2.03 (0.42)
FEV_1_ post-bronchodilator (%pred)	63.0 (8.7)	59.6 (9.9)	59.9 (9.8)	62.5 (9.5)
FEV_1_/IVC% post-bronchodilator	48.7 (8.9)	44.2 (8.9)	44.3 (9.5)	47.7 (8.6)
Geometric mean methacholine PC_20_ (mg/ml)	0.76 (2.9)	0.39 (3.0)	0.67 (1.9)	0.41 (2.4)

Patient characteristics for current smokers and ex-smokers with COPD and groups treated with placebo and fluticasone (only compliant patients). Bronchial biopsies were available at baseline of 64 (elastic fibers), 56 (versican), 61 (decorin), 61 (collagen I) and 64 (collagen III) patients. After 30 months, bronchial biopsies of 32 compliant patients were available, tissue from 29 (elastic fibers), 26 (versican), 27 (decorin), 28 (collagen I) and 28 (collagen III) patients had sufficient surface area for analysis (≥0.09 mm^2^) (fluticasone and placebo groups combined). Data are presented as mean (SD) or median (IQR), unless otherwise stated. Methacholine PC_20_: provocative concentration of methacholine that causes a 20% decrease in FEV_1_, expressed as mean doubling doses. Part of the data have been published previously [17], [22], [27].

* p<0.05 compared to current smokers (two tailed unpaired t-tests).

### Inhaled Corticosteroids and Extracellular Matrix Proteins

**Figure 2:**
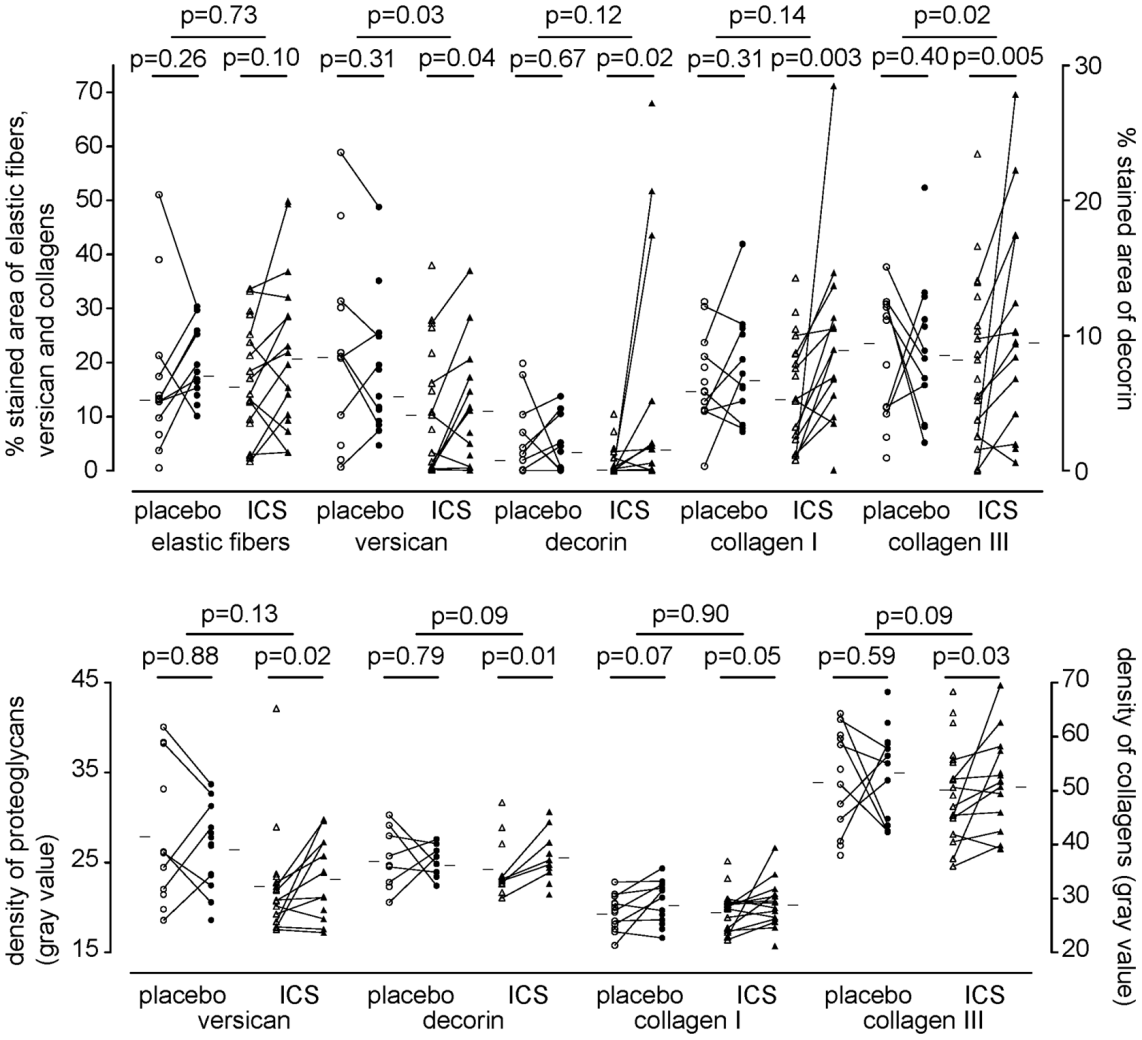
Percentage and density of stained area for placebo and fluticasone for all ECM proteins. Percentage (upper panel) and density (lower panel) of stained area in bronchial biopsies is presented. Open figures: baseline percentage stained area, closed figures: percentage stained area after 30 months. Horizontal bars represent medians.

**Figure 3:**
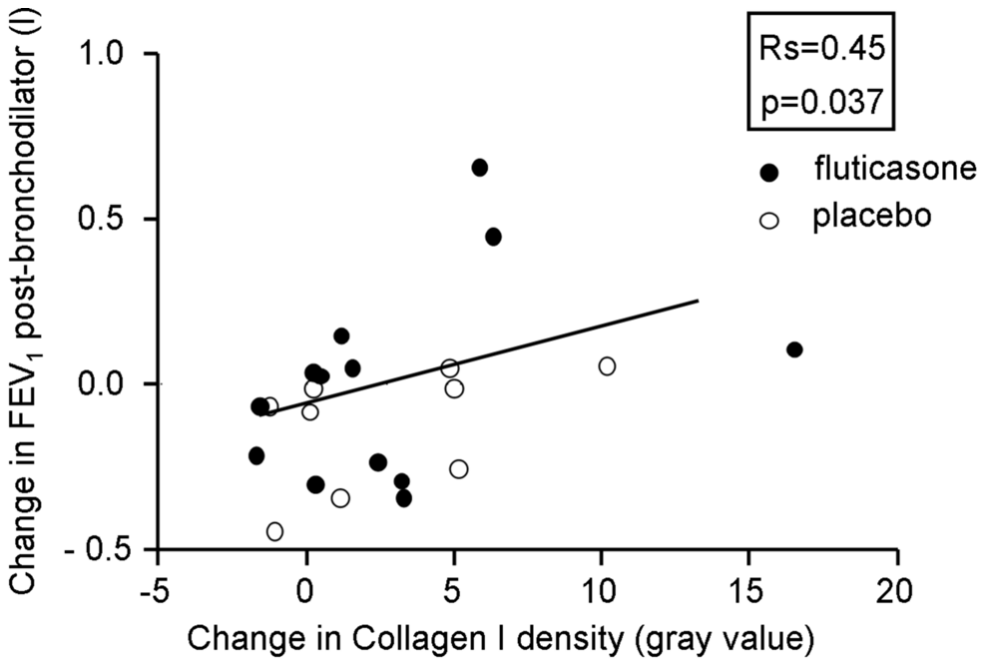
Correlation between change in post-bronchodilator FEV_1_ (L) and change in density of collagen I. Both values represent values after 30 months minus values at baseline. Closed circles represent fluticasone treated subjects, open circles represent placebo treated subjects.

Adjusted for baseline values, we found that ICS significantly increased percentage versican (9.6% [CI 0.9 to 18.3%]; p = 0.03) and collagen III (20.6% [CI 3.8 to 37.4%]; p = 0.02) compared to placebo (figure 2); a trend was seen for the density of decorin (3.9 [CI −0.7 to 8.6]; p = 0.09) and collagen III (8.4 [CI −1.1 to 17.9]; p = 0.09). Baseline percentage and density of versican (17% [CI 3.5 to 30.6%]; p = 0.02 and 8.0 [CI 2.7 to 13.3]; p = 0.006, respectively) and collagen III (10.7% [0.1 to 21.4%]; p = 0.03 and 7.9 [CI 0.9 to 15.0]; p = 0.05, respectively) and percentage of decorin (2.0% [CI 0.5 to 3.5%]; p = 0.02) were significantly higher in the placebo group than the fluticasone group. Change in smoking status was not included into our analysis, because current and ex-smokers with COPD had similar ECM composition. An increase in density of collagen I was associated with improvements in post-bronchodilator FEV_1_ (l) (Rs = 0.45, P = 0.037) when we analyzed both fluticasone and placebo treated groups combined (figure 3). No correlations were found for other ECM proteins and lung function.

### Smoking Status and Extracellular Matrix Proteins at Baseline

**Figure 4:**
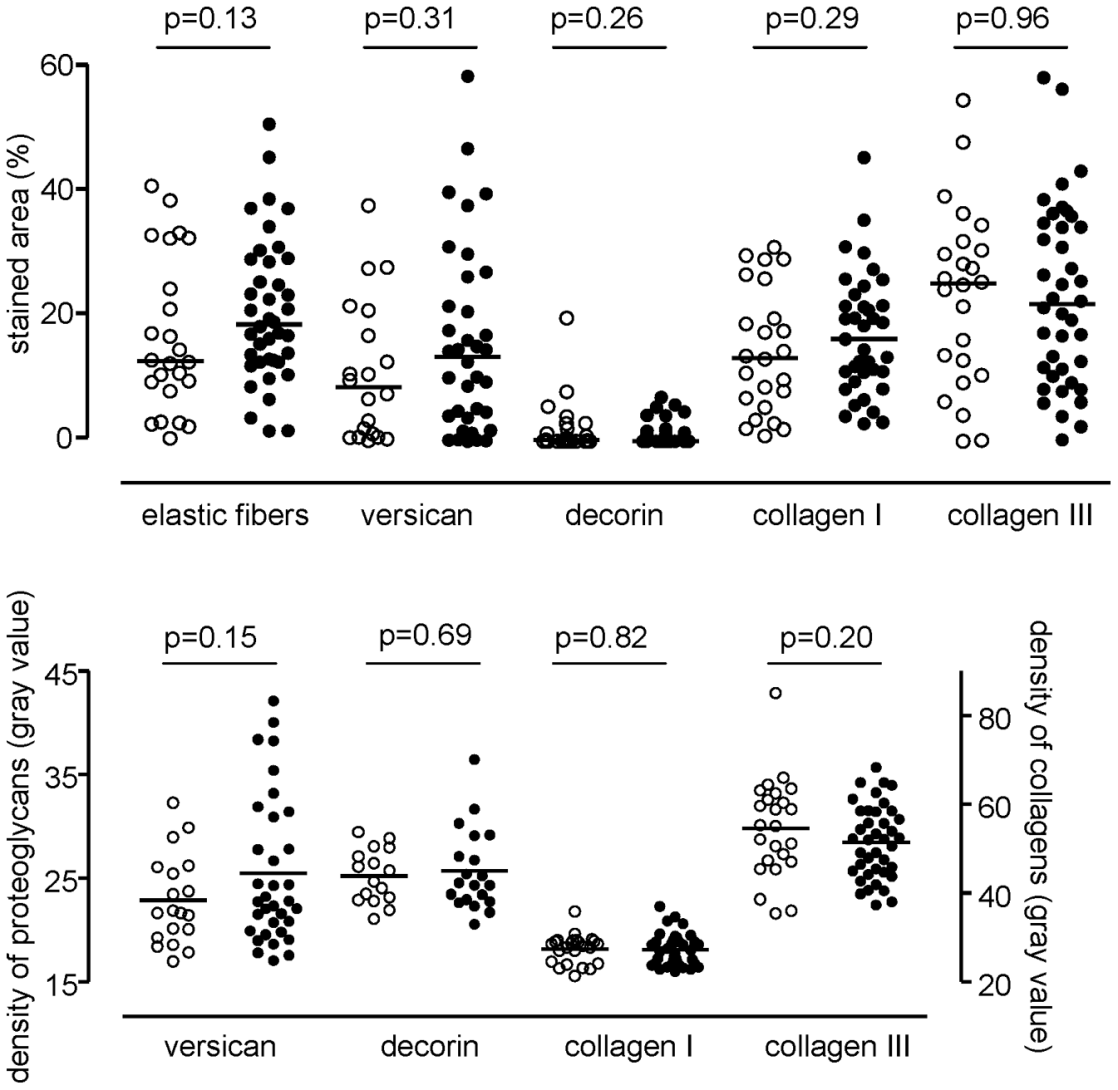
Percentage and density of stained area at baseline of ex-smokers and smokers with COPD. Percentage (upper panel) and density (lower panel) of stained area in bronchial biopsies is presented. Ex-smokers are presented as open circles, current smokers as closed circles. Horizontal bars represent medians. No significant differences were found for all studied extracellular matrix proteins (both percentage stained area and density).

No significant differences in percentage of the area being positively stained and density of ECM proteins were found between current smokers and former smokers with COPD (figure 4). Long-term ex-smokers (≥5.5 years, our median value) had similar percentage and density of all ECM proteins compared to short term ex-smokers (<5.5 years) and current smokers (all P>0.05). Furthermore, no relation was found between packyears and percentage or density of all ECM proteins.

### Correlations between Extracellular Matrix and Lung Function at Baseline

**Figure 5:**
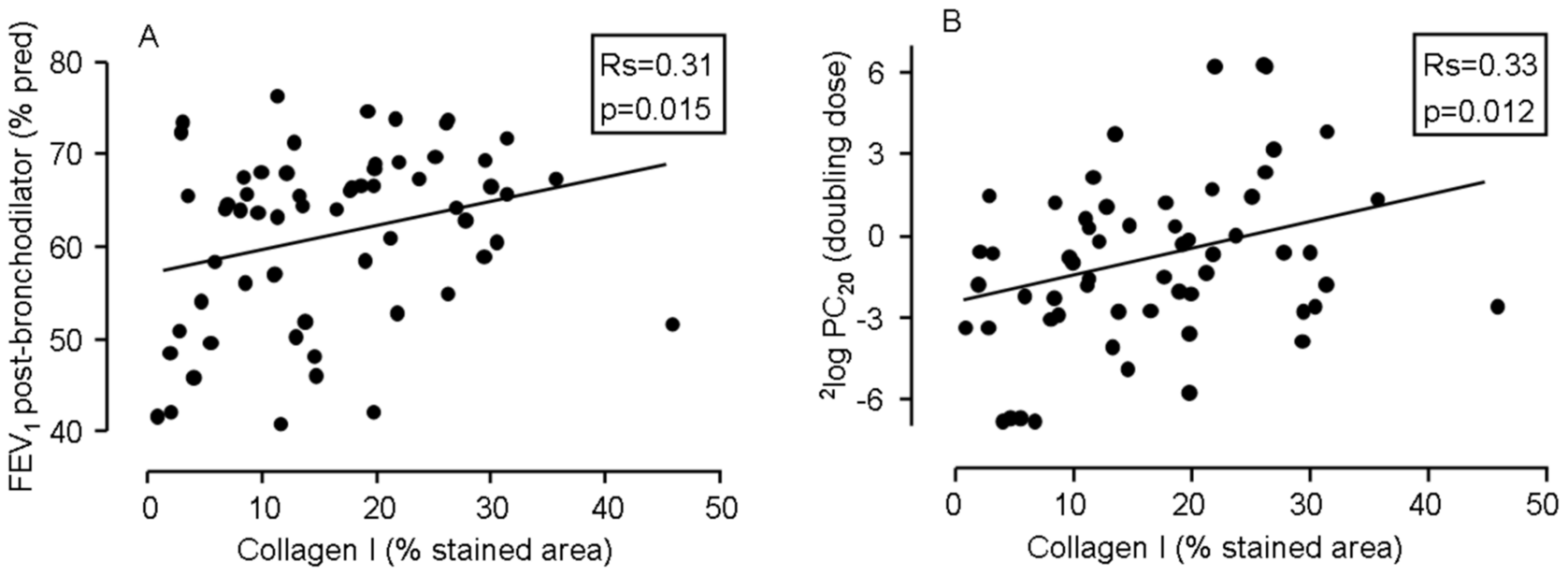
Correlation between percentage collagen type I at baseline and lung function parameters. Panel A presents post-bronchodilator FEV_1_ (% predicted) and panel B shows PC_20_ (in doubling dose).

Percentage collagen I correlated positively with FEV_1_ (% predicted) post-bronchodilator (Rs = 0.31, P = 0.015) (figure 5, left panel) and FEV_1_/IVC% (Rs = 0.38, P = 0.003). In addition, percentage collagen type I and III correlated with PC_20_ (Rs = 0.33, P = 0.012; Rs = 0.37, P = 0.004, respectively) (figure 5, right panel). Percentage collagen I, but not collagen III, was significantly lower in GOLD stage III (n = 9) than GOLD stage II (n = 55) (medians 5.5% and 17.7%, respectively, P = 0.01). No significant correlations were found between lung function at baseline and densities of all ECM proteins.

## Discussion

Our results show that 30-month treatment with inhaled corticosteroids increases the percentage stained area of versican and collagen III, indicating that long-term treatment possibly influences the remodeling process in the airways. Furthermore, lung function is weakly, but positively correlated with collagen I both at baseline and with regard to changes in FEV_1_ and collagen I that occurred after treatment. In addition, we show that the content of ECM proteins in bronchial biopsies did not significantly differ between smokers and ex-smokers with moderate-severe COPD.

This study shows that the content of elastic fibers, major proteoglycans and collagens in the bronchial mucosa are similar in current and ex-smokers with COPD. Our findings extend previous observations, showing no difference in the percentage elastic fibers in COPD patients and smokers without airway obstruction [11], [28]. We observed no significant difference in versican and decorin content between current and ex-smokers with COPD, which is in line with an *in vitro* study with cultured lung fibroblasts of moderate COPD patients and control subjects. Cigarette smoke extract (CSE) exposure of these cells did not affect versican gene expression, but decreased decorin gene expression [16]. This apparent difference with our findings could be explained by the fact that smoke-exposed fibroblasts are only selectively triggered compared to a multifactorial environment *in vivo*. Finally, in our study collagen type I and III were not significantly different between current and ex-smokers with COPD, which is similar to recent observations in cultured fibroblasts of COPD and non-COPD patients [29].

The percentage of versican and collagen III increased with long-term ICS treatment compared to placebo, without significant changes in elastic fibers, decorin and collagen I. In line with this, ICS for four weeks or 3.5 years did not affect elastic fibers content in bronchial biopsies of asthmatics compared to healthy controls [18]. Notably, we found a significant increase in collagen III, but not collagen I, after 2.5 years of ICS treatment compared to placebo, which was associated with lung function. Previous studies in COPD patients showed that gene expression of collagen 1α1 and collagen 3α1 in small airways and parenchyma was decreased in association with lower FEV_1_ [3], [30]. Thus, collagen may have stabilizing effects on the collapsible airways in patients with COPD, which could be further enhanced by long-term use of ICS.

Our study has various strong points. We included only steroid-naive COPD patients, excluding possible influences of steroids on ECM components at baseline. Both the percentage and density of the stained area in bronchial biopsies were analyzed: the percentage corresponds to the presence of the ECM protein, whereas density represents the local amount of ECM protein. For the analysis of the percentage, we used the total selected tissue area for analysis as an internal reference parameter according to the recommendations of the Joint ATS/ERS Task Force [24]. We considered the possibility that part of our changes is explained by an effect of ICS on edema. However, less edema resulting from ICS treatment would probably have increased percentage and density all studied ECM proteins, whereas in our study the percentage of only some ECM proteins was affected. Furthermore, we previously found lower numbers of selected bronchial inflammatory cells after ICS treatment in the current study [17]. We did not find correlations between the effect of ICS treatment on inflammatory cells and ECM components (data not shown).

There are some considerations when interpreting our results. Matched bronchial biopsies both at baseline and follow-up were available from approximately half of our COPD patients, because part of the tissue was no longer available. This could have negatively affected the power of our study. Still, the number of available biopsies was similar among both groups. Furthermore, since one biopsy per patient per visit was studied, we cannot exclude that local heterogeneity of ECM proteins has affected our results. To minimize selection bias, we only selected biopsies with the largest lamina propria. Lung tissue specimens from healthy or never-smokers were not available, but comparisons with these groups were beyond the objectives of this study. Furthermore, features of remodeling in COPD are different between large and small airways, nevertheless we evaluated the ECM in the central airways only [2] and important correlations with lung function could still be observed. Finally, despite treatment randomization, we accidentally found that the percentage and densities of versican, decorin and collagen III at baseline were significantly higher in the placebo than the fluticasone group. Not withstanding this, when still adjusted for the baseline values, we observed effect of ICS therapy. Taken together, we do not believe that the above limitations largely affected our results.

How can we explain that smoking has no effect on ECM? Exposure of cultured pulmonary fibroblasts of moderate and very severe COPD patients to CSE resulted in downregulation of decorin, but not versican and collagen type I and III expression [16], [29]. In addition, collagen I and tropoelastin were dose-dependently inhibited by CSE in rat fetal lung fibroblasts [31]. Mice with long-term exposure to cigarette smoke showed a decrease in elastic fibers and collagen type III, without significant effect on collagen I [32]. We could only partially confirm these *in vitro* and animal studies. Inflammation and remodeling in the lung *in vivo* are simultaneous and complex ongoing processes and may not be mimicked by studies in isolated fibroblasts and inbred animals kept under specific conditions. Furthermore, after smoking cessation bronchial inflammation (at least) partially persists [22], which is in line with our finding of similar ECM composition between smokers and ex-smokers.

We showed a positive correlation between the content of collagen and lung function after treatment with inhaled steroids. However, the current opinion of remodeling is that airway wall thickening is strongly associated with progression of COPD [2], suggesting that increased ECM deposition is related to a decreased lung function. How can we explain this apparent contradiction? In COPD, an imbalance between proteases and anti-proteases is present, as shown by an excess of matrix metalloproteinases (MMP) and a relative shortage of tissue inhibitor of metalloproteinases (TIMP) [33]. MMP degrade both collagens and proteoglycans [2], [33], [34]. Dexamethasone can reduce MMP-9 and increase TIMP-1 release from alveolar macrophages of COPD patients [35], which may result in a decreased capacity to degrade ECM. This is in line with our observation that ICS increase collagen and versican. Also the observation from Annoni *et al* [11], showing that patients with COPD have lower collagen I densities in their airways, is in line with the speculation that an increase in collagen I induced by ICS could stabilize the airways. Furthermore, the observed positive correlation between collagen with lung function and PC_20_ before and after long-term ICS therapy also suggests that increased airway wall fibrosis is actually preventing both airway collapse and attenuating airway smooth muscle contractions in COPD. Besides airway remodeling, emphysema might also influence airway collapse, which could contribute to the airflow obstruction. Unfortunately, no data were collected to quantify the extent of emphysema in our cohort of COPD patients.

Although smoking cessation shows positive clinical effects [1], smoking status was not significantly correlated with our studied ECM components. Treatment with ICS increased the percentage versican and collagen III. We found positive correlations between ECM proteins and several lung function parameters at baseline and after treatment with ICS. Therefore, our data may implicate that steroids alter airway structure by increasing ECM content in COPD which is associated with preserved lung function. This suggests that increased presence ECM proteins do not by themselves lead to detrimental consequences, but instead can prevent airway collapse.

In conclusion, we showed that treatment for 30 months with inhaled corticosteroids increased the relative content of versican and collagen III in the large airways of patients with moderate to severe COPD. Our data suggest that steroids not only prevent bronchial inflammation but possibly also alter airway structure by increasing specific ECM proteins in COPD that are associated with improvements in lung function. Further studies are needed to confirm these findings in other studies, and to understand the possible implications of these findings for current treatment strategies and for the development of future, targeted anti-remodeling medication in COPD.

## Supporting Information

Figure S1 **Study flow diagram.** Study flow diagram of the GLUCOLD study presenting the bronchial biopsies used in this study at baseline and after 30 months treatment with inhaled fluticasone.
(TIF)

Protocol S1 Trial protocol GLUCOLD study.
(DOC)

File S1 (DOC)

Checklist S1 CONSORT checklist.
(PDF)
