# Supplementary figures and images for: Inhaled Steroids Modulate Extracellular Matrix Composition in Bronchial Biopsies of COPD Patients: A Randomized, Controlled Trial
Source: PLoS One. 2013 May 7;8(5):e63430. doi: 10.1371/journal.pone.0063430 (PMC3646783; doi:10.1371/journal.pone.0063430)

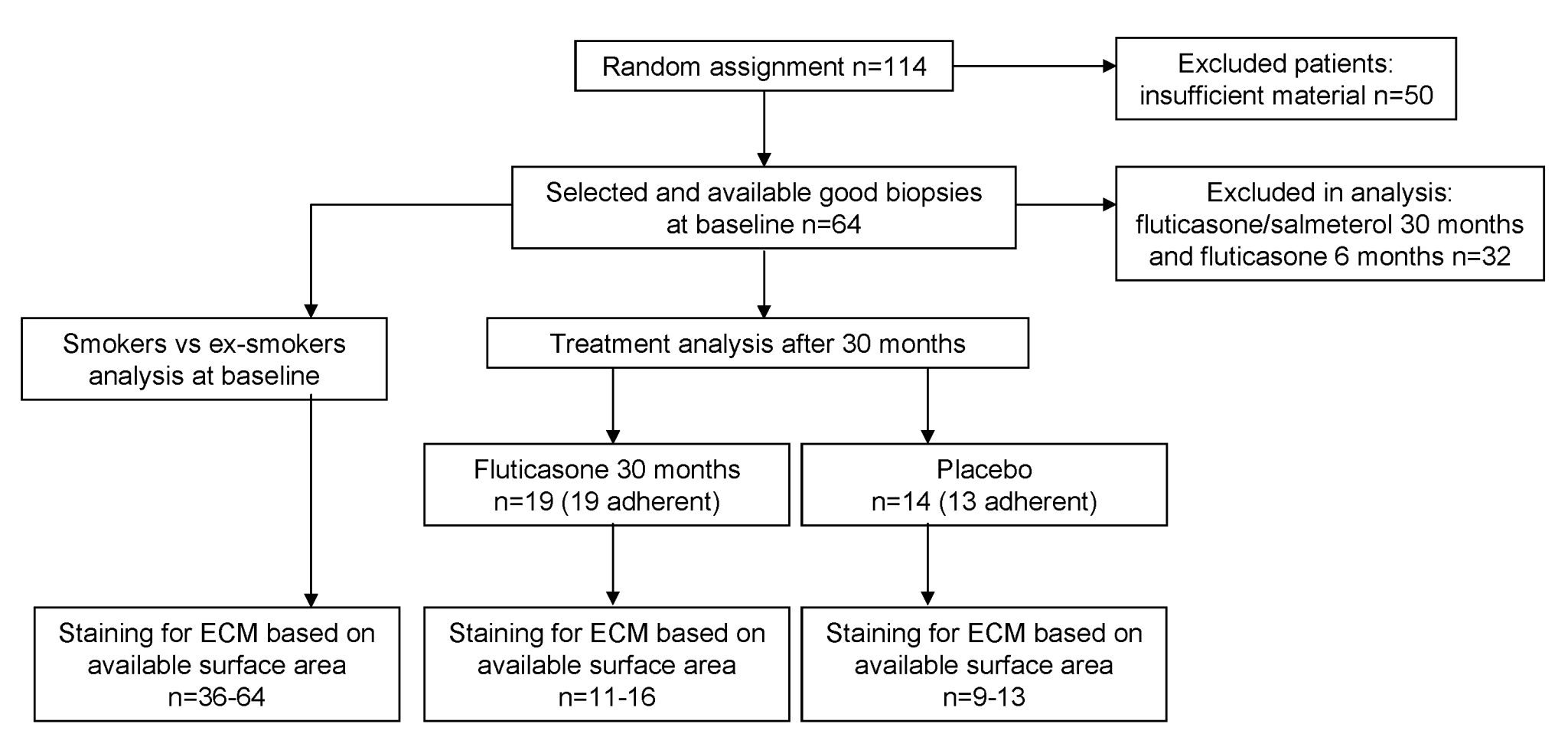

Supplement: Figure S1 — Study flow diagram. Study flow diagram of the GLUCOLD study presenting the bronchial biopsies used in this study at baseline and after 30 months treatment with inhaled fluticasone. (TIF) [file pone.0063430.s001.tif]
