# Supplementary material for: Inhaled Steroids Modulate Extracellular Matrix Composition in Bronchial Biopsies of COPD Patients: A Randomized, Controlled Trial
Source: PLoS One. 2013 May 7;8(5):e63430. doi: 10.1371/journal.pone.0063430 (PMC3646783; doi:10.1371/journal.pone.0063430)
Supplement: File S1 — (DOC) [file pone.0063430.s003.doc]

**Supplementary Material**

**Inhaled steroids modulate extracellular matrix composition in bronchial biopsies of COPD patients: a randomized, controlled trial**

Lisette I.Z. Kunz, Jolanda Strebus, Simona E. Budulac, Therese S. Lapperre, Peter J. Sterk, Dirkje S. Postma, Thais Mauad, Wim Timens, Pieter S. Hiemstra and the GLUCOLD (Groningen Leiden Universities Corticosteroids in Obstructive Lung Disease) Study Group.

**MATERIAL and METHODS**

**(Immuno)histochemical stainings**

Sections of 4 μm thickness of paraffin-embedded bronchial biopsies were used for histochemistry (elastic fibers) and immunohistochemistry (proteoglycans, collagens). All sections were deparaffinized in xylene and rehydrated in ethanol and demineralized water. Elastic fibers were histochemically stained according to Weigert’s protocol [24]. Sections were incubated in equal amounts of Weigert’s solution 1 and 2 (Klinipath, Duiven, The Netherlands) for 10 minutes and washed in tap water. Then the sections were heated for 30 seconds by a microwave oven in Weigert’s resorchin-fuchsin stain (Klinipath) and left at room temperature (RT) for 1.5 hours, which was done twice. Excess stain was rinsed off in 95% ethanol and washed in water. Van Gieson stain (Klinipath) was incubated for one minute, and the sections were dehydrated in ethanol. Elastic fibers stained by Van Gieson stain appear black.

Antigen retrieval was used for all immunohistochemical stainings (table S1). Sections were heated in citrate solution pH 6.0 (DAKO, Glostrup, Denmark) at 97°C for 30 minutes and cooled on ice. Chondroitinase ABC (Sigma-Aldrich, St Louis, MO, USA) 0.05 U/ml was incubated for one hour at 37°C. Trypsin 0.1% (T7409, Sigma-Aldrich) and calcium chloride (CaCl_2_) 0.1% (Sigma-Aldrich) were dissolved in Tris-HCl 0.05M (pH 7.6) and sections were incubated for 20 minutes at 37°C. All washing steps were performed with phosphate buffered saline (PBS). All primary antibodies were diluted in PBS with 1% bovine serum albumin (BSA) and incubated for one hour or overnight (decorin) at RT. The horseradish peroxidase (HRP)-conjugated anti-mouse or anti-rabbit EnVision system (DAKO) was used for detection of versican, decorin and collagen III. For Collagen I, a rabbit-anti-goat HRP-conjugated antibody (DAKO) was incubated for 30 minutes (dilution 1:100), followed by HRP conjugated anti-rabbit EnVision system (DAKO). NovaRed (Vector, Burlingame, CA, USA) was applied as a chromagen for all IHC stainings and sections were counterstained with Mayer’s hematoxylin (Klinipath). All glasses were mounted with Pertex mounting medium (HistoLab, Gothenburg, Sweden). As negative controls, mouse IgG1, rabbit Ig-fraction and goat serum were used for mouse, rabbit and goat primary antibody, respectively (all DAKO), followed by protocol described above. Images of stained biopsies are presented in figure 1 in the manuscript.

**Digital image analysis**

Tissue samples were analyzed in a blinded manner by independent observers, unaware of the subjects’ clinical data (LK for elastic fibers and proteoglycans; JS for collagens). Total biopsy images were acquired using a color camera (200x magnification) and analyzed with image analysis software (CellD, Olympus, Zoeterwoude, The Netherlands). The lamina propria was selected per biopsy (minimum area 0.09 mm^2^), including the reticular basement membrane, but excluding large blood vessels, mucous glands and airway smooth muscle and damaged tissue. The colors of the stained area were separated following the color model ‘CMYK’ (Cyan, Magenta, Yellow, Black). To obtain optimal detection and contrast, the Y-phase was used for the immunohistochemical stainings (thresholds 18-100), the K-phase for the elastic fibers (thresholds 90-164). The detection limit of stained area used for analysis was at least 100 pixels (=140 μm^2^). The percentage stained area was calculated dividing the stained area by the total selected area. The mean selected area in which the percentage stained area was calculated, was 4.5 (elastic fibers), 3.5 (versican), 3.3 (decorin), 4.4 (collagen I) and 5.6 mm^2^ (collagen III). Staining intensity was further analyzed by densitometry (weighted mean per biopsy) and presented as gray value (black: gray value=0; white: gray value=255). Only immunohistochemical stainings can represent density, therefore density was not calculated for elastic fibers.

**Table S1: Primary antibodies used for immunohistochemical stainings.**

| Antibody | Antigen retrieval^*^ | Species | Dilution | Incubation time | Origin^†^ |
| --- | --- | --- | --- | --- | --- |
| Collagen I | Citrate pH 6.0 | Goat | 1:400 | 1 hour | US Biologicals |
| Collagen III | Trypsin | Mouse | 1:1000 | 1 hour | Oncogene &Calbiochem |
| Versican | Trypsin | Mouse | 1:1000 | 1 hour | Seikagaku |
| Decorin | Chondroitinase ABC | Rabbit | 1:800 | Overnight | Gift P.J. Roughley |

^*^ Citrate pH 6.0, DAKO, Glostrup, Denmark; Trypsin, Sigma-Aldrich, St. Louis, MO, USA; Chondroitinase ABC, Sigma-Aldrich.

^†^ US Biologicals, Swampscott, Massachusetts; Oncogene &Calbiochem, Darmstadt, Germany; Seikagaku corporation, Tokyo, Japan.
